# Case Report: SMART ANTON: Anton-Babinski Syndrome in Stroke-Like Migraine Attacks (SMART) After Radiation Therapy: Two Rare Syndromes, One Case

**DOI:** 10.3389/fneur.2022.887287

**Published:** 2022-06-27

**Authors:** Nicolas Nagysomkuti Mertse, René Müri

**Affiliations:** ^1^Department of Neurology, University Hospital Bern, Bern, Switzerland; ^2^Department of Psychiatry, University Hospital of Psychiatry and Psychotherapy, University of Bern, Bern, Switzerland; ^3^Translational Research Center, University Hospital of Psychiatry and Psychotherapy, University of Bern, Bern, Switzerland

**Keywords:** SMART syndrome, Anton-Babinski syndrome, dual etiology, Riddoch syndrome, visual associative agnosia, cortical blindness

## Abstract

**Introduction:**

We describe the case of a 57-years-old patient who presented an Anton-Babinski syndrome in the context of a stroke-like migraine attack after radiation therapy (SMART).

**Case Report:**

The patient was brought to the emergency room following a sudden loss of vision in the context of a pre-existing left-sided hemianopia after excision of a right occipital astrocytoma followed by radio-chemotherapy 35 years prior to his admission in our services. At admittance, he also presented hyperthermia, hypertension, and a GCS of 7. The MRI showed a leptomeningeal enhancement in the left temporal, parietal, and occipital lobes. After exclusion of other differential diagnoses, we diagnosed a cortical blindness in the context of a SMART syndrome affecting the left hemisphere. While the symptoms improved under corticosteroid therapy, the patient successively presented an Anton-Babinski syndrome, a Riddoch syndrome and a visual associative agnosia before finally regaining his usual sight.

**Discussion:**

This is, to our knowledge, the first report of an Anton-Babinski syndrome in the context of a SMART syndrome. A dual etiology is mandatory for cortical blindness in SMART syndrome since the latter affects only one hemisphere. A SMART syndrome affecting the contralateral hemisphere in respect to the radiation site seems to be uncommon, which makes this case even more exceptional.

## Introduction

The Anton-Babinski syndrome (ABS) is a rare phenomenon observed in few cases of cortical blindness characterized by the lack of awareness for the visual deficits (anosognosia) and vivid confabulations (1). It was first described in 63 AD by the roman politician Seneca as he depicted the strange behavior of his wife's blind maid who kept denying her blindness (2). The name was given centuries later as a tribute to the neurologists Gabriel Anton and Joseph Babinski for their research in related fields (3, 4).

The Stroke-Like Migraine Attacks after Radiation Therapy (SMART) syndrome is a rare late-onset complication of brain radiation therapy characterized by a unilateral cortical gadolinium enhancement typically associated with seizures and stroke-like episodes with prolonged reversible symptoms (5).

While the Anton-Babinski syndrome has previously been described in the context of radiation-induced leukoencephalopathy (6), this is, to our knowledge the first report of an Anton-Babinski syndrome in the context of a Stroke-like Migraine Attack after Radiation Therapy.

## Case Report

The 57-year-old patient's neurological history started 35 years prior to his admission to our hospital with the surgical excision of an astrocytoma in the right occipital lobe followed by a local radio-chemotherapy (Carmustine, 54 Gy) and a subsequent permanent left sided hemianopia. The patient was hospitalized 3, 12 and 21 years after this treatment following a sudden transient cortical blindness, left-sided occipital headache, meningeal signs, disorientation, sensory aphasia, and a right sided hemiparesis. Since the patient also presented fever, he was treated with antibiotics and virostatics at least once, though no evidence of viral or bacterial infection could be objectified. No evidence in favor of an autoimmune genesis, a status epilepticus or a posterior reversible encephalopathy syndrome was found. Twenty years after resection, the patient developed a focal status epilepticus and was put under antiepileptic treatment with valproate and phenobarbital. The treatment was switched to levetiracetam 1,000 g/day and lamotrigine 50 g/day following a valproate encephalopathy 9 years after treatment initiation. For the past 5 years prior to his admission, the patient had remained seizure free.

**Figure 1:**
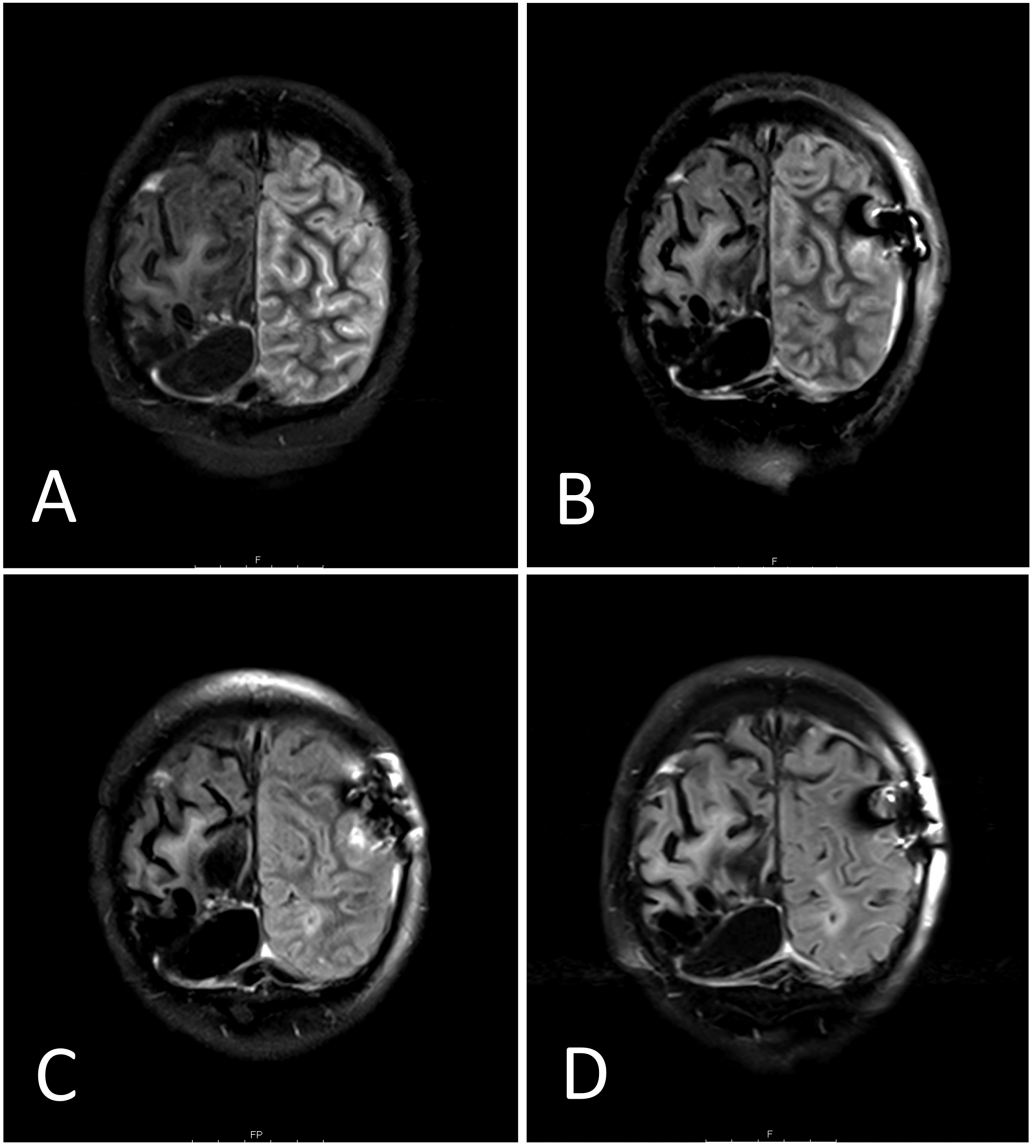
Side to side comparison MRI flair day 1 **(A)**, day 6 **(B)**, day 14 **(C)** and day 45 **(D)**. The follow-up MRIs shows a gradual reduction of the initially displayed leptomeningeal enhancement in the left temporal, parietal and occipital lobes. The substance defect after astrocytoma excision appears as a liquid-filled black cyst in the lower part of the right hemisphere (left-sided in the pictures).

The current hospitalization occurred following a sudden loss of vision followed by a comateous state according to the patient's wife. He was initially announced by the paramedic services with a GCS of 7, hyperthermia and hypertension and had been given 1,000 mg levetiracetam and 3 mg midazolam on suspicion of a seizure considering his medical history. MRI showed signs of a pronounced left-sided temporo-parieto-occipital meningoencephalitis with cortical blood-brain barrier damage, leptomeningeal enhancement as well as a small left-sided parieto-occipital infarction (Figure 1). The patient regained consciousness within 2 h following admission. The neurological exam showed an anomic aphasia, disorientation as well as a discrete weakness of the right hand.

An EEG at admission showed theta-dominant background activity with recurrent mostly bifrontal generalized rhythmic delta activity, as well as a moderate focal slowing of the posterior left hemisphere and a mild slowing focus with a breach effect of the posterior left hemisphere without signs of ongoing epileptic activity or spreading of the bifrontal delta activity.

A cerebrospinal fluid analysis showed a slight pleocytosis (10 cells /mm^3^) as well as elevated protein (1.43 g/L) and lactate (2.7 mmol/L) levels. Recurrent microbiology analysis of both cerebrospinal fluid and blood showed no sign of infection or autoimmune condition. A cerebral biopsy was performed in the left parietal lobe, where immunohistochemistry objectified the presence of disseminated single CD4-, CD8- and CD3-positive T-cells and CD68-positive macrophages. No signs of IgG-mediated disease (B-cells), neoplastic lesion or infection were found.

After exclusion of other potential etiologies, a stroke-like migraine attack after radiation therapy (SMART) was diagnosed. Although there was no evidence of epileptic activity on admission nor in following EEGs, we suspected, based on the initially displayed symptoms (decreased awareness, fever, hypertension), that a seizure might have occurred beforehand, either as a symptom or as a trigger of the SMART syndrome. We therefore increased the patient's previous seizure prophylaxis to 3,000 mg levetiracetam per day. We also initiated a steroid therapy starting with a steroid pulse therapy with intravenous methylprednisolone 1 g per day for 3 days followed by a switch to a body-weight adapted oral steroid therapy with prednisolone 100 mg per day. The latter was gradually reduced while the patient's symptoms steadily improved. Neuroradiology confirmed a continuous regression of the inflammation under this treatment. After 4 weeks at the acute care unit, the patient was transferred to our neurorehabilitation department. At admission, he presented an Anton-Babinski syndrome as well as a discrete residual right-sided hemiparesis and was still receiving 50 mg prednisolone per day. The neuropsychological assessment at admission revealed a severe formal thought disorder including logorrhea, tangential speech, loose association, perseverations, semantic and phonematic paraphasia and neologisms. We also found severe mnestic deficits, a reduced alertness, and mild deficits in verbal fluency.

**Figure 2:**
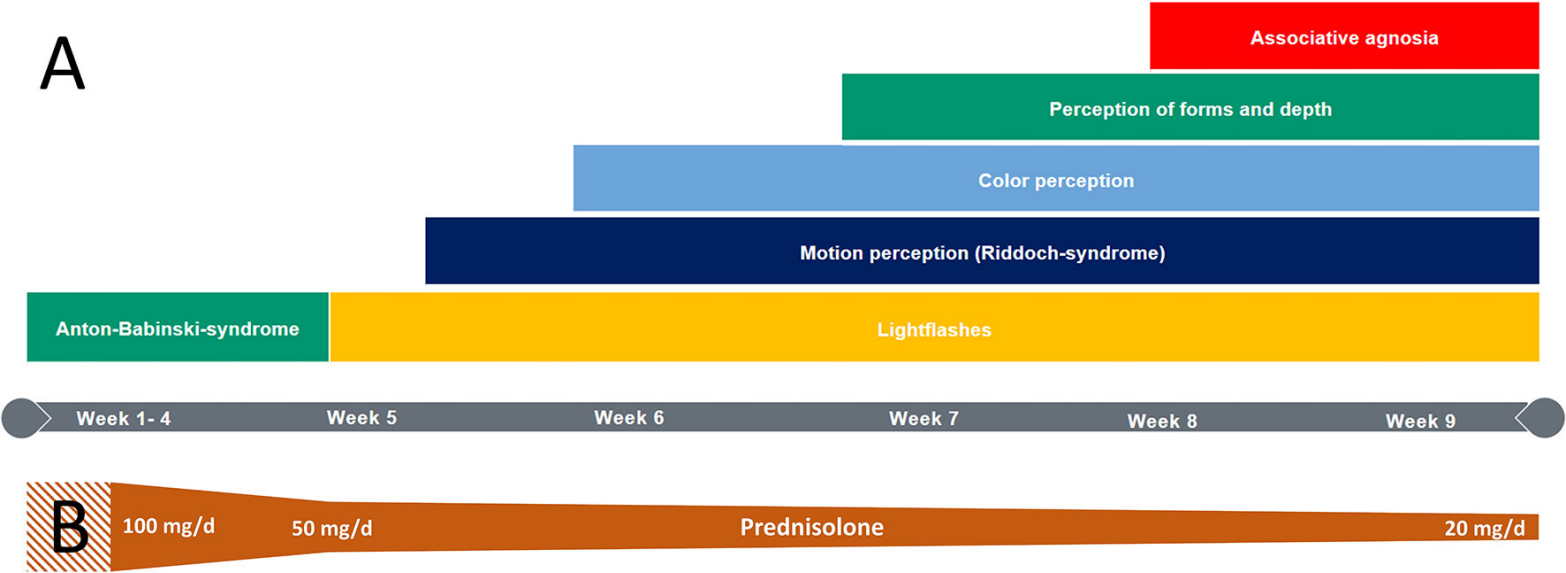
**(A)** Visual perception recovery during hospitalization (right hemifield); **(B)** Visualization of the treatment with corticosteroids: Steroid pulse therapy with 1 g intravenous methylprednisolone during the first 3 days (striped pattern) followed by oral therapy with prednisolone 100mg gradually reduced over 9 weeks.

During the patient's 6-week stay in neurorehabilitation, the steroid therapy was gradually reduced from 50 to 20 mg prednisolone per day. His symptoms rapidly improved under this treatment (Figure 2). On the third day after admission to neurorehabilitation, he was able to perceive and react to flashlights. After a week, he began perceiving rapid hand movements. Interestingly, motion perception was objectified in both hemifields despite the preexisting left-sided hemianopia after partial occipital resection. Counting up to three fingers was possible when kept in motion. After another week, the patient began perceiving colors in his right hemifield and could describe shapes on the same side another week later. Eight to nine weeks after symptom onset, the patient merely showed a slightly impaired visual acuity in the right hemifield (beside the pre-existing left sided hemianopia after astrocytoma excision). Accordingly, a distinct regression of the inflammation was found in MRI (Figure 1).

Although the patient's eyesight had mostly recovered, we noticed that he had troubles naming objects. While he could describe them, he could name the objects only after touching them. We therefore diagnosed a visual associative agnosia, which eventually resolved 2 weeks later, after which he was transferred to another hospital.

While the patient was glad to have recovered his eyesight and hence his independence in most of the basic activities of daily life, he persisted on never having been blind and blamed his temporary vision problems on an inadequate correction of his glasses.

## Discussion

### Diagnosis and Treatment of the SMART Syndrome

Black et al. (7) proposed revised diagnostic criteria including a typical patient profile (history of irradiation, no signs of residual or recurrent neoplasm), typical neuroimaging findings (transient unilateral cortical gadolinium enhancement) with a matching typical symptomatology (e.g., seizure, headache, unilateral motor, sensory or visual deficits) and the exclusion of other potential underlying causes. Our patient's case, with a history of a known cerebral irradiation in the context of astrocytoma in 1985, the displayed prolonged but mostly reversible neurological symptoms (anomic aphasia, motor and visual deficits) many years after irradiation, the massive unilateral band-shaped gadolinium uptake in MR diagnostics and finally the lack of evidence for another cause of the symptomatology, largely meets these criteria with one atypical feature though: the affected hemisphere was not the one that had been primarily targeted 35 years earlier. It is partly because of this untypical feature that a cerebral biopsy was performed in order to exclude other differential diagnoses, although it has been pointed out as potentially harmful and doubtfully useful for the diagnostic of a SMART syndrome (5, 8). The contralateral affection in our case may in fact be related to an incidental co-irradiation of the contralateral hemisphere due to technical targeting inaccuracy.

The precise mechanism of pathogenesis of the SMART syndrome is still unclear. It has been discussed whether it might be triggered by epileptic seizures acting as a second hit on a brain already pre-damaged by a previous radiation therapy which highlights the importance of sufficient seizure prophylaxis (5). The blood-brain barrier disruption and the subsequent brain oedema objectified via gadolinium enhancement makes the use of steroids seem sensible and may speed up symptom regression, although full recovery has also been described without the use of steroids (8).

### SMART-Related Cortical Blindness and Its Dual Etiology

Cortical blindness describes a specific form of blindness involving a bilateral functional impairment of the visual cortex. The SMART syndrome, on the other hand, is a radiologically unilateral condition (7), which implies that a dual etiology is mandatory for the occurrence of a SMART-related cortical blindness. In our case, the preexisting functional impairment consisted in a tissue defect in the right occipital lobe following an astrocytoma excision 35 years earlier.

As mentioned above, the hemisphere affected by the SMART syndrome in our patient was not the one targeted during radiation therapy. A contralateral affection has been described before (7) but seems to be rather uncommon according to larger case series (9, 10), which makes our case even more exceptional.

### Cortical Blindness With Residual Motion Perception

While our patient slowly regained his eyesight, we noticed that he could perceive motions in both hemifields despite the preexisting left-sided hemianopia after partial occipital resection. Since the SMART syndrome only affected his left hemisphere, we assume that the residual motion perception in the left hemifield was unaffected by the SMART syndrome but had been overlooked at admittance due to the severe formal thought disorder and the confabulations initially displayed by the patient. Residual motion perception can indeed be found in ABS (1). In our case, the residual motion perception is likely related to the right visual area V5, which is critical for motion perception (11) and had been spared during the excision of the astrocytoma. V5 has direct connections to the posterior thalamus and superior colliculus (12) allowing visual information to bypass the primary visual cortex (PVC) and directly access V5 for further processing, eventually leading to motion perception in the blindfield of patients with an ipsilateral damaged PVC, a phenomenon called Riddoch syndrome found in many cases of hemianopia and sometimes in cortical blindness (13, 14).

### Anton-Babinski Syndrome: Confabulations, Anosognosia and the Global Network Theory

The emergence of confabulations and anosognosia has been discussed for quite some time, a much-cited paper from 1989 by McGlynn and Schacter (15) offers several potential explanations. In the light of some particular features of our case, we considered Bernhard J. Baar's Global Workspace Theory on conscious perception of stimuli for some interesting alternative theoretical approaches.

According to the Global Workspace Theory (GWT) proposed by Baars et al. competing perceptual information from the cortices form coalitions based on the internal consistency of the information they convey (16). The strongest coalition induces a “winner-take-all” equilibrium by broadcasting its information to other cortical areas, hence creating an internally consistent information stream that rises into consciousness. This theoretical background gives rise to the following approaches on the emergence of confabulations and anosognosia in ABS:

#### V5

A damage to the PVC leads to a disruption of the feedback-loop between the PVC and the thalamus, which means that neither the lack nor the existence of visual perception is reported. A residual motion perception in cortical blindness conveyed by a still functional area V5 like in our case may, based on the principle “winner-take-all” and without opposing information from the PVC, suggest an intact visual perception to consciousness, ultimately resulting in anosognosia. The perception of a stimulus thus suggested combined with the missing ability to fully perceive this same stimulus eventually creates an internal conflict when the patient tries to produce a verbal description of what he sees. This conflict can ultimately only be solved by the unconscious fabrication of an imaginary description, in short: a confabulation.

#### Parietal Stream

The distinction of relevant information from irrelevant requires an considerable computational effort (17). The mammalian brain addresses this problem by using the global workspace to broadcast only relevant information into consciousness recruiting cortical resources, while irrelevant information is processed unconsciously in specialized and localized brain areas (18). These so-called “frames” in which relevant visual stimuli are interpreted are part of the parietal stream and are critical for the integration of visual perception into consciousness (16). A disruption of these frames disturbs the conscious visual perception and can lead to a neglect of visual input in the contralateral hemifield. Cases of bilateral neglect have been reported (19). Patients presenting both a neglect and a hemianopia are often unaware of their visual deficit, which is why the distinction between the two is challenging at times (15, 20). While neglect is most common in afflictions of the parietal lobe (e.g., middle cerebral artery stroke), it has also been described in posterior cerebral artery strokes in association with damage to white matter regions of the occipital lobe (21). Accordingly, a patient with a bilateral occipital damage under involvement of underlying white matter regions may be blind and at the same time neglect both hemifields, hence neglecting his blindness and simultaneously be anosognostic, a mechanism also discussed by McGlynn et Schacter, though in the context of hemianopia (15). This hypothetical approach does not entirely match our case though. While a right-sided negect could be related to the involvement of the left parietal lobe [since per definition the SMART syndrome only affects gray matter regions (7)], a left-sided neglect would have to be related to the occipital white matter damage after tumor excision and would hence be permanent, which was not the case.

## Limitations

This case report has a few limitations. First, the visual field was not measured with objective methods (e.g., perimetry), the described improvements are hence solely based on clinical observation. Second, while the SMART syndrome is a probable diagnosis, it remains a diagnosis of exclusion (7), another etiology may have been overseen. Finally, the confabulations could also be interpreted in the context of the initial severe formal thought disorder, the latter would not sufficiently explain the persistent anosognosia for the cortical blindness though.

## Conclusion

This is, to our knowledge, the first report of an Anton-Babinski syndrome with a dual etiology, but also of an Anton-Babinski syndrome in the context of a SMART syndrome. A dual etiology is required for an Anton-Babinski syndrome to occur in the context of a SMART syndrome. The Global Workspace Theory offers interesting approaches to understand the underlying mechanism of confabulations and anosognosia in Anton-Babinski syndrome.

## Data Availability Statement

The original contributions presented in the study are included in the article/supplementary material, further inquiries can be directed to the corresponding author.

## Ethics Statement

Written informed consent was obtained from the individual(s) for the publication of any potentially identifiable images or data included in this article.

## Author Contributions

NN collected the clinical data and wrote the first draft of the paper. RM supervised the work and corrected the paper several times during the drafting process. All authors approved the final version.

## Funding

Open access funding was provided by the University of Bern.

## Conflict of Interest

The authors declare that the research was conducted in the absence of any commercial or financial relationships that could be construed as a potential conflict of interest.

## Publisher's Note

All claims expressed in this article are solely those of the authors and do not necessarily represent those of their affiliated organizations, or those of the publisher, the editors and the reviewers. Any product that may be evaluated in this article, or claim that may be made by its manufacturer, is not guaranteed or endorsed by the publisher.
