# Erratum: Case report: SMART ANTON: Anton-Babinski syndrome in stroke-like migraine attacks (SMART) after radiation therapy: Two rare syndromes, one case

**DOI:** 10.3389/fneur.2023.1182387

**Published:** 2023-03-21

**Authors:** Frontiers Production Office

**Affiliations:** Frontiers Media SA, Lausanne, Switzerland

**Keywords:** SMART syndrome, Anton-Babinski syndrome, dual etiology, Riddoch syndrome, visual associative agnosia, cortical blindness

An omission to the funding section of the original article was made in error. The following sentence has been added: “Open access funding was provided by the University of Bern.”

The publisher apologizes for this error. The original article has been updated.

